# Association of fructose consumption with prevalence of functional gastrointestinal disorders manifestations: results from Hellenic National Nutrition and Health Survey (HNNHS)

**DOI:** 10.1017/S0007114523001198

**Published:** 2023-12-14

**Authors:** Theodoros Smiliotopoulos, Antonis Zampelas, George Houliaras, Spiros N. Sgouros, George Michas, George Bamias, Demosthenes Panagiotakos, Nikolaos Cholopoulos, George P. Chrousos, Eleftheria Roma, Emmanuella Magriplis

**Affiliations:** 1 Laboratory of Dietetics and Quality of Life, Department of Food Science and Human Nutrition, Agricultural University of Athens, 11855 Athens, Greece; 2 First Department of Pediatrics, Medical School, National and Kapodistrian University of Athens, 11527 Athens, Greece; 3 Department of Gastroenterology, Athens Naval Hospital, 7011528 Athens, Greece; 4 Department of Nutrition and Dietetics, School of Health Science and Education Harokopio University, 17676 Athens, Greece; 5 Department of Medicine, School of Health Sciences, Aristotle University of Thessaloniki, University Campus, 54124 Thessaloniki, Greece; 6 University Research Institute of Maternal and Child Health and Precision Medicine and UNESCO Chair on Adolescent Health Care, Medical School, National and Kapodistrian University of Athens, 11527 Athens, Greece

**Keywords:** Functional gastrointestinal disorders prevalence, Irritable bowel syndrome, ROME III, Fructose consumption, Mediterranean diet

## Abstract

The study aimed to assess the total prevalence of functional gastrointestinal disorders (FGID), and separately, irritable bowel syndrome (IBS) among adults and to determine their potential association with fructose consumption. Data from the Hellenic National Nutrition and Health Survey were included (3798 adults; 58·9 % females). Information regarding FGID symptomatology was assessed using self-reported physician diagnosis questionnaires the reliability of which were screened using the ROME III, in a sample of the population. Fructose intake was estimated from 24 h recalls, and the MedDiet score was used to assess adherence to the Mediterranean diet. The prevalence of FGID symptomatology was 20·2 %, while 8·2 % had IBS (representing 40·2 % of total FGID). The likelihood of FGID was 28 % higher (95 %CI: 1·03–1·6) and of IBS 49 % (95 %CI: 1·08–2·05) in individuals with higher fructose intake than with lower intake (3rd tertile compared with 1st). When area of residence was accounted for, individuals residing in the Greek islands had a significantly lower probability of FGID and IBS compared with those residing in Mainland and the main Metropolitan areas, with Islanders also achieving a higher MedDiet score and lower added sugar intake, comparatively to inhabitants of the main metropolitan areas. FGID and IBS symptomatology was most prominent among individuals with higher fructose consumption, and this was most conspicuous in areas with a lower Mediterranean diet adherence, suggesting that the dietary source of fructose rather than total fructose should be examined in relation to FGID.

Gastrointestinal symptoms^(1)^ are quite common, often qualifying as functional gastrointestinal disorders (FGID), due to the frequent recurrence and chronic nature of the complaints mainly attributed to the pharynx, oesophagus, stomach, biliary tract, intestines or anorectal area^(2)^. These health conditions include various symptoms, such as heartburn, gastroesophageal reflux disease, dyspepsia/indigestion, nausea and vomiting, gas, bloating and irritable bowel syndrome (IBS)^(3)^; all of which cause major discomfort and frequently result in work absenteeism^(4)^. FGID is a serious issue for health providers and a major burden on health services across the globe since reports have shown that an average of 40 % of the total human population is affected^(5)^. At the same time, in West European countries, 20–50 % of FGID symptomatology is attributed to IBS^(2)^, leading to an intensive search for the main risk factors contributing to total FGID symptomatology and IBS specifically, with diet having been implicated to FGID symptomatology. Of course, gut dysbiosis, the condition of reduced bacterial diversity and imbalance of the gut microbiota,^(6)^ stands among the other dietary, genetic, lifestyle, psychological and environmental factors, and FGID occurrence, as a potential cause or consequence of the FGID symptomatology, with a clear distinction between them yet to be discovered^(7,8)^. Consequently, gut dysbiosis, which may lead to gut dysmotility and visceral hypersensitivity, is often believed to be part of the pathophysiological mechanism of the FGID occurrence and is targeted by pharmaceutical and dietary interventions^(9)^.

Overall, fructose consumption has been associated with many chronic diseases, such as nonalcoholic fatty liver disease, CVD diabetes^(10,11)^ and FGID, with great attention being given to the latter^(12)^ due to its high prevalence. Latest studies have focused on fermentable oligosaccharides, disaccharides, monosaccharides and polyols and FGID onset or relief^(4,13–15)^ with results being highly controversial with respect to disease onset. Fructose is widespread in fresh and processed foods; it is found in small quantities in fruits, vegetables and pulses, in conjunction with fibre, and in large quantities in processed food, such as sugar sweetened beverages. Some studies have correlated fructose intake with IBS^(16,17)^ mostly due to fructose malabsorption, whereas other studies failed to show such an association^(18,19)^. A case–control study that assessed differences in the habitual diet between individuals with IBS and healthy controls found that cases had a higher fat and lower fructose and fibre intakes^(20)^ compared with controls, although it was not clear whether findings were due to reverse causality, meaning that cases may had removed fructose- and fibre-containing foods from their diet due to their symptoms. Another case–control study failed to find any differences among consumers of various food groups in patients with total FGID compared with healthy controls^(21)^. Further adding to this controversy, a recent study showed that a higher adherence to the Mediterranean Diet – relatively high in fructose from fruits, vegetables and pulses – resulted in a decrease in FGID prevalence^(18)^. The Western diet is a dietary pattern also relatively high in fructose, but it differs significantly from the Mediterranean diet, since it contains a large percentage of highly processed food, which are the main sources of fructose in this dietary pattern^(22)^. Specifically, the Western diet is characterised by high fructose corn syrup sweetened beverages and fruit drinks (often sweetened with apple juice, which has a fructose to glucose ratio higher than high fructose corn syrup – 2·2:1), juices or nectars, which provide large proportions of free and added fructose^(23)^, whereas fructose from the Mediterranean diet is derived from whole fruit and vegetables and legumes^(24,25)^. At this point, it is important to mention that high fructose corn syrup (characterised as isoglucose or glucose–fructose syrup in the EU) is commonly used in the USA with a fructose to glucose ratio exceeding 1:1 going sometimes over the generally recognised as safe 55 % fructose^(26,27)^, whereas in the EU glucose–fructose syrups contain significantly lower amounts of fructose ranging from 5 % to 50 %^(28–30)^. However, EU has recently relaxed prior restrictions on import of high fructose corn syrup, and hence the higher ratio can be the one used in food manufacturing^(31)^. Western diet foods have been implicated as proinflammatory that may increase risk of IBS^(32)^. These results raise the question whether total fructose intake induces and/or enhances FGID symptoms or whether the foods that are rich sources of it also play a role.

Therefore, the aim of the present study was to primarily examine FGID prevalence and its association with fructose consumption using data from a national nutrition and health study of the Greek population. Prevalence of IBS symptomatology, a specific FGID, was further evaluated in relation to fructose intake.

## Materials and methods

### Study design

Data from the Hellenic National Nutrition and Health Survey (HNNHS) were used to define prevalence and associated dietary and socio-economic factors of gastrointestinal disorders in general with an additional focus on IBS symptomatology. The HNNHS followed a multistage stratified design, based on age, sex and area of residence provided by the Hellenic Statistical Authority (2011 Census)^(33)^. It took place from 1 September 2013 to 31 May 2015 and collected health and dietary data of non-institutionalised individuals ≥ 6 months, living in Greece. Non-Greek-speaking citizens, pregnant and lactating women, servants of the armed forces, institutionalised individuals and people unable to provide informed consent (unless a first-degree relative assisted) were excluded from the study. Details regarding sampling and design have been already described elsewhere^(33)^. Briefly, a multistage sampling stratification was performed by region, sex and age group. The final sample was representative by geographical area as follows: mainland (21·8 % of sample), islands (9·6 % of sample) and the two major municipal centres (Attica and Central Macedonia; 68·6 % of the sample). Data collection was performed via standardised in-home interview by trained personnel. Sampling details and distributions have been previously published^(33)^. For the current study, data from a total of 3·798 (40·6 % males) Greek adults were used.

The Ethics Committee of the Department of Food Science and Human Nutrition of the Agricultural University of Athens and the Hellenic Data Protection Authority approved the study, and in addition, all staff members signed confidentiality agreements and all adult participants signed a consent form to participate.

### Dietary assessment

The methods used in HNNHS for the dietary assessment are in accordance with the European Food Safety Authority’s recommendations for the harmonisation of data across member state countries of the European Union^(34)^. In summary, two 24 h recalls of non-consecutive days were aimed to be collected from participants using the USA Department of Agriculture’s^(35)^ Automated Multiple Pass Method^(36)^. The first was derived through Computer Assisted Personal Interview method and the second by telephone with the help of validated food atlases and photographs of standardised household measures (cups, grids and plates). These were used to accurately determine portion sizes, since the pictures corresponded to specific grams of food reported and selected, during the first interview and a copy was given to the participants to use during the phone interview that followed, with details are described elsewhere^(33)^. In summary, the nutritional value of all foods and drinks consumed was calculated, using the Nutrition Data System for Research, a Windows-based dietary analysis program designed by the Nutrition Coordinating Center at the University of Minnesota, for the collection and analyses of 24-hour dietary recalls, food records, menus and recipes^(37)^, and the mean intake of the two days was then calculated for the estimation of total energy and macronutrient intakes. Extreme over- and under-reporters were excluded (*n* 102; < 600 kcal per day and > 6000 kcal per day, respectively). Total sugars were differentiated from mean carbohydrate intake. Total sugars are defined as the sum of all free mono and disaccharides’ including glucose, fructose, galactose, lactose as well as sucrose and maltose. For the purpose of this paper, total fructose was subtracted from total sugar intake in order to assess the effect of their intake on FGID (and IBS specifically), while adjusting for other sugar intake^(21)^. Added sugar, defined as ‘all sugars and syrups added to foods during processing or preparation, excluding those naturally found in food’ was also computed. All macronutrients were computed in relation to individual mean energy consumption (% total energy). Finally, the MedDiet score was calculated to address the influence of the Mediterranean diet on FGID. The MedDiet score includes eleven food components that well describe the Mediterranean diet composition. The final score ranges from 0 to 55, with 0 being no adherence and 55 being perfect adherence to the Mediterranean diet pattern^(38)^. Details regarding the MedDiet score calculation have been provided elsewhere^(39)^.

### Gastrointestinal symptoms

Data were collected through a valid self-reported Medical History Questionnaire^(33,40)^. Specifically, individuals were asked whether they had been previously diagnosed with any FGID condition by a physician, such as gastroesophageal reflux disease, IBS or any other abdominal discomfort. The Greek official translation of the ROME-III questionnaire for adults designed for clinical practice and research was used in a random sample of the study population that consented to assess reliability of self-response. ROME III is a validated method used in a clinical setting to determine the presence of functional gastrointestinal disorders^(41)^. ROME III and not IV was used because the latter had not yet been developed when HNNHS study was conducted^(42)^. ROMEIII, however, remains valid since a recent meta-analysis found that this may be less suitable for epidemiological surveys, due to the more restrictive criteria it employs^(43)^. The Greek version of the ROME III questionnaire was developed following the ROME foundation’s official guidelines and can be accessed through the foundation’s official website (https://theromefoundation.org/). Results from ROME III were categorised as percent individuals with any FGID, and percentage of IBS specifically.

**Fig. 1.**
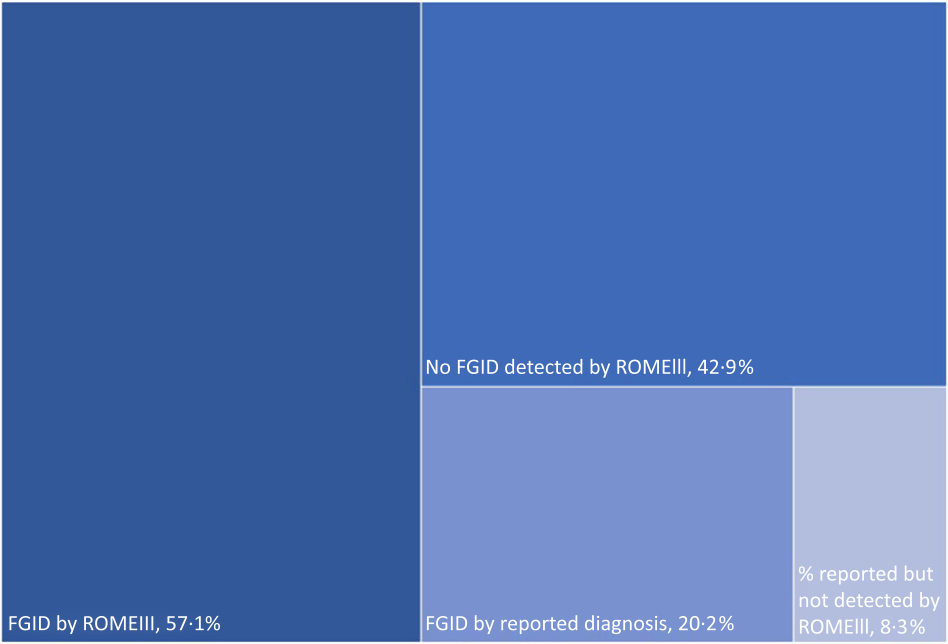
Hierarchy graph of FGID distribution by ROME III and/or reported by diagnosis. FGID by ROMEIII: percent individuals found to have at least 1 functional gastrointestinal disorder (FGID) based on the ROME Association criteria. FGID by reported diagnosis: percent of individuals that reported having been diagnosed with at least one type of FGID by a physician. % Reported but not detected by ROMEIII: percent individuals that reported having been diagnosed by a physician but not detected by ROMEIII.

To assess the validity of self-reporting FGID status, a sensitivity analysis was conducted (Fig. 1) comparing results obtained from both questionnaires: ROMEIII questionnaire and questions pertaining to having been diagnosed (test of detected *v*. reported FGID condition). All individuals that were detected by ROME III questionnaire as having at least one FGID had also reported having been diagnosed by their physician, while a remaining 8·3 % of the total population had reported being diagnosed by a physician, but were not detected by ROMEIII (Fig. 1). Based on the high correlation between physician diagnosis and ROMEIII categorisation, data from self-reported condition were used in the analysis to estimate FGID prevalence and its association with fructose intake accounting for other socio-demographic, lifestyle and dietary parameters (reported below). Prevalence of IBS specifically was evaluated separately due to high prevalence of this bowel disorder^(21,43)^.

### Socio-demographic and lifestyle data

Various types of socio-demographic and lifestyle characteristics were collected through Computer Assisted Personal Interview, including sex, age, marital and employment status. Information on lifestyle data including sleeping (mean hours per day) and smoking habits (current-, ex-, never- smoker) and coffee and alcohol consumption frequency were reported. Mental health was assessed by evaluating the presence of depressive symptoms using the Patient Health Questionnaire-9, and further details have been provided elsewhere^(44)^. Reported weight (in kg) and height (in metres) were used to calculate BMI using the equation weight/height^2^ (kg/m^2^), and the participants weight status was categorised according to the WHO categorisation^(45)^. Due to the borderline low (none < 17 kg/m^2^) and very small percentage of underweight individuals, these were grouped with those of normal weight and are used to describe the sampled population. For further analysis, overweight and obese individuals were also grouped creating a binary variable for weight status (adults with healthy weight and those with overweight or obesity)^(46)^. The International Physical Activity Questionnaires adapted for adults and for elderly^(47)^ was used to estimate the physical activity (PA) level of the participants. According to the questionnaire’s results, all participants were categorised in four categories (sedentary, light, moderate or high PA). Sedentary activity included individuals not meeting the light PA category.

### Statistical methodology

Survey design analysis was used to present socio-demographic and lifestyle characteristics, weighted by area (according to sampling structure and the 2011 Hellenic population census), and categorical variables are presented as relative frequencies. Continuous variables were tested for normality of their distribution using P-P and k-density plots and were presented as mean ± standard deviation^(35)^ for normally distributed and as median and quartiles (50 (25 – 75)) for those skewed. Between-group differences were tested using Wilcoxon rank-sum test (for skewed variables), two-sample *t* test (for normally distributed variables) or Pearson *χ* ^2^ test (for categorical variables). Pearson *χ* ^2^ tests were used to determine between category distribution differences, and adjusted Wald test was performed post hoc to determine within-groups differences. Although multiple tests were performed, raw p values have been included. Multiple logistic regression models were used to estimate the odds of at least one FGID and IBS specifically with tertile of fructose consumption. Specifically, two models were used: one minimally adjusted for age and sex and another fully adjusted for all a priori known variables associated with FGID and those found to differ significantly between presence or non-presence of FGID condition during preliminary analysis. The final model included fructose consumption, sex, age (per 5-year increase), marital status, saturated fats consumption, depression and smoking status derived from the preliminary analysis and MedDiet score categories and energy intake as important factors intervening to the outcome. Post estimations of IBS manifestation were performed by area, due to significant geographical variations (main metropolitan areas, Islands & Crete and remaining Mainland). Multicollinearity was checked for all the predictors in all models via variance inflation factor and Spearman’s rank correlation. The absence of multicollinearity between the predictors can be accepted when the variance inflation factor is < 10, and no moderate or strong correlation is found between covariates^(48–50)^ (hence all *r* ≤ 0·39). Predicted probability of IBS was depicted in relation to mean MedDiet score and added sugar intake for each area (descriptive analysis, secondary to aims). Significance was set at alpha = 5 %. Database cleaning and statistical analysis were performed using the statistical package STATA 17.0 (StataCorp, Texas ltd.).

## Results

Of 3·798, 765 participants, 20·1 % experienced an FGID condition, 8·2 % of whom were diagnosed with IBS specifically (40·1 % of those with FGID) (Table 1). Self-reports on physician’s diagnosis regarding the presence of FGID were used in conjunction with the results based on ROME III questionnaire as the two methods were found to be highly correlated, as explained in Methods (Fig. 1). A higher prevalence of FGID and/or IBS was detected in females, in adults aged 50 years or more years, and among divorced/separated or widowed individuals (*P* for all < 0·001). Also, individuals living in the two main Metropolitan areas of Greece (Attica and Central Macedonia) had the highest proportion of FGID and IBS conditions whereas individuals living in islands the lowest. Lastly, significant difference in FGID (and IBS specifically) was found within total family salary status, with individuals reporting at least two salaries having significantly lower prevalence.

**Table 1.** Socio-demographic characteristics in the study population by gastrointestinal (GI) and IBS status

	Underlying FGID symptom			Underlying IBS symptom		
	% per specific category*	% from total population†	*P* value*	%per specific category*	% from total population†	*P* value†
Total *n* 3798		20·2 %		40·1 %	8·2 %	
Sex			< 0·001			< 0·001
Females‡,§	21·8	12·8		10·2	6·0	
Males‡,§	13·3	5·4		4·4	1·8	
Age (years)			< 0·001			0·003
19–30‡,§	14·7	4·8		6·1	2·0	
31–50‡,§	16·6	5·3		7·6	2·4	
51+‡,§	21·1	7·5		7·0	2·5	
Geographical area			< 0·001			< 0·001
Attica and Thessaloniki‡,§	22·4	15·3		9·0	6·1	
Islands and Crete‡,§	11·6	1·1		3·4	0·3	
Mainland‡,§	14·4	3·1		5·8	1·2	
Total family salary status			0·003			0·249
One salary‡,§	16·0	5·4		6·8	2·3	
At least one pension‡,§	21·4	4·1		6·3	1·2	
No salary‡,§	18·6	3·8		7·9	1·6	
One salary and at least one pension‡,§	20·6	1·5		9·9	0·7	
At least two salaries‡,§	14·6	2·7		5·7	1·1	
Marital status			< 0·001			0·025
Married/cohabit‡,§	17·2	7·4		6·7	2·9	
Divorced/separated‡,§	31·1	1·4		19·9	0·9	
Single‡,§	16·2	6·9		6·9	2·9	
Widowed‡,§	23·5	1·7		7·1	0·5	
Educational family level||			0·926			0·139
Higher education including colleges	18·0	9·7		7·5	4·1	
12 years of school	16·8	5·0		7·0	2·1	
Up to 6 years of school	17·6	1·6		3·4	0·3	
≥ 2 members with higher education	19·4	1·4		8·4	0·6	

Values are weighted for population distribution and living area with primary sampling unit (PSU): household (family or another bond under the same household). Weighted percentages (%) are depicted for total population and percentages by variable of interest. *P* values are based on Pearson *χ* ^2^ tests for within groups total difference. Total *n* of participants for each category not shown due to the use of the svy: stata command to obtain shown percentages. Significance set at alpha = 5 %.

Raw *P* values are provided. Type I error following Bonferroni correction, at 5 % level, is n/0·05, which is approximately < 0·0007; a *P* value < 0·001, although significant, is not precise to conclude.

* Existence of at least one FGID or IBS symptom, based on Rome III diagnostic questionnaire (*n* 168) and self-reports in HNNHS (*n* 3630) between each specific category (e.g., %FGID symptoms between female participants).

† Existence of at least one FGID or IBS symptom, based on Rome III diagnostic questionnaire (*n* 168) and self-reports in HNNHS (*n* 3630).

‡ Significant differences within groups for individuals with FGID symptomatology.

§ Significant differences within groups for individuals with IBS symptomatology.

||Summary of the educational level of a family as a whole.

Table 2 presents the weight status and lifestyle characteristics of the study population by total FGID symptoms and IBS status. A significant higher prevalence of any FGID symptom and IBS specifically was found in individuals (i) with sleeping problems, (ii) with chronic stress, (iii) with depression and (iv) in current and ex-smokers (*P*_for all_ < 0·001). When FGID prevalence was assessed for each one of the aforementioned statuses, it was found two to three times higher among those experiencing the addressed conditions (35·9 % for individuals with sleeping problems compared with 15·6 % for those without; 43·1 % for individuals with chronic stress compared with 14·5 % for those without and 30·9 % for individuals with depression compared with 16·3 % for those without). On the other hand, individuals with high PA levels had fewer FGID symptoms compared with those having sedentary or low PA levels.

**Table 2.** Anthropometric and lifestyle characteristics of the study population by gastrointestinal (GI) status

	Underlying FGID symptom					Underlying IBS symptom				
	% per specific category*			% of total population	*P* value*	% per specific category*			% of total population	*P* value†
		Mean	sd				Mean	sd		
BMI (kg/m2), Mean†		25·8	5·2		0·177		25·4	5·2		0·334
Weight status†										
Normal weight	17·7			8·5	0·082	7·7			3·7	0·567
Underweight	14·1			0·4		5·2			0·1	
Overweight	16·8			5·3		6·0			1·9	
Obese	19·4			3·0		7·3			1·1	
Sleeping hours					0·208					0·298
< 6 h	48·3			0·5		17·2			0·2	
≥ 6, < 8 h	56·1			2·0		10·1			0·4	
≥ 8 h	44·2			1·0		9·6			0·2	
Sleeping problems‡,§					< 0·001					< 0·001
No	15·6			13·6		5·8			5·0	
Yes	35·9			4·4		16·6			2·0	
Alcohol consumption					0·700					0·456
No	17·7			4·8		6·0			1·6	
Yes	17·6			12·7		7·3			5·3	
Chronic Stress‡,§					< 0·001					< 0·001
No	14·5			12·4		5·4			4·6	
Yes	43·1			5·5		19·2			2·5	
Depression‡,§					< 0·001					< 0·001
No	16·3			14·3		6·4			5·6	
Yes	30·9			3·4		12·9			1·4	
Smoking					< 0·001					0·021
Never smoked‡,§	15·1			7·5		6·0			3·0	
Current smoker‡,§	20·5			6·8		8·2			2·7	
Ex-smoker	19·6			3·1		7·3			1·2	
MedDiet score||					0·748					0·233
< 23	16·7			2·7		5·7			0·9	
≥ 23	18·0			13·9		7·4			5·7	
IPAQ score					0·029					0·398
Very active PA	15·9			6·1		6·4			2·5	
Moderate PA	17·1			6·4		6·6			2·5	
Sedentary-low PA	20·5			2·2		7·8			1·6	

FGID, functional gastrointestinal disorders; IBS, irritable bowel syndrome.

Values are weighted for population distribution and living area with primary sampling unit (PSU): household (family or another bond under the same household). Weighted percentages (%) are depicted for total population and percentages by variable of interest. *P* values are based on Pearson *χ* ^2^ tests for within groups total difference or *t* test based on survey design. Total *n* of participants for each category not shown due to the use of the ‘svy’ stata command to obtain shown percentages.

Raw *P* values are provided. Type I error following Bonferroni correction, at 5 % level, is n/0·05, which is approximately < 0·0007; a *P* value < 0·001 is not precise to conclude.

* Existence of at least one FGID or IBS symptom, based on Rome III diagnostic questionnaire or (*n* 326) and HNNHS (*n* 3630) between each specific category (e.g. %FGID symptoms between female participants).

†BMI categorised based on WHO categorisation (underweight: BMI < 18·5 kg/m^2^ – normal weight: 18·5 kg/m^2^ ≤ BMI < 25 kg/m^2^ – overweight: 25 kg/m^2^ ≤ BMI < 30 kg/m^2^ – obese: BMI ≥ 30 kg/m^2^).

‡ Significant differences within groups for individuals with FGID symptomatology.

§ Significant differences within groups for individuals with IBS symptomatology.

||MedDiet score cut-off selected based on population median.

Macronutrient intake by FGID and by IBS specifically is presented in Table 3. Mean fructose intake was higher among individuals with both FGID and IBS with no observed difference within each tertile in terms of FGID symptomatology. Added sugars intake was higher among those with IBS than those without, within the 1st tertile of added sugars consumption. Mean total PUFA and total sugars intake (excluding fructose) were also higher in individuals with FGID symptomatology than those without. Sugars specifically were also higher within the 2nd tertile of their consumption for individuals with IBS than those without (*P*_for all_ < 0·050). No other ‘raw’ significant differences were observed between groups.

**Table 3.** Mean population macronutrient consumption* by total FGID and IBS status, using 24 h recall

	FGID symptoms					IBS				
	Mean daily consumption*					Mean daily consumption*				
Macronutrient*	FGID symptoms		No FGID symptoms			IBS		No IBS		
	Mean/Median	sd/25th–75th percentile	Mean/Median	sd/25th–75th percentile	*P*-value1 †	Mean/Median	sd/25th–75th percentile	Mean/Median	sd/25th–75th percentile	*P*-value2 ‡
Fats^6^, % total energy	37·9	10·6	38·0	10·6	0·729	38·0	10·5	38·0	10·6	0·979
MUFA	16·8	6·3	17·1	6·3	0·235	16·9	5·9	17·1	6·4	0·595
PUFA	5·1	3·9–6·7	4·9	3·8–6·3	0·015	5·1	3·9–6·6	4·9	3·3–6·4	0·065
Saturated fats	12·4	4·3	12·6	4·4	0·374	12·5	4·5	12·5	4·4	0·873
Protein^6^, % total energy	13·5	6·4	13·6	6·3	0·615	13·3	6·2	13·6	6·3	0·499
Animal protein	6·6	3·7–10·7	7·0	3·8–11·3	0·132	6·3	3·7–10·8	7·0	3·7–11·2	0·253
Vegetable protein	4·8	3·4–7·0	4·8	3·3–6·8	0·376	4·8	3·5–7·0	4·8	3·3–6·8	0·352
Carbohydrates^6^, % total energy	12·2	3·8	12·0	3·7	0·188	12·3	3·7	12·1	3·8	0·370
Total sugars (without fructose), % total energy	9·6	6·1–14·3	9·2	5·5–14·0	0·007	10·6	6·2–14·7	9·1	5·5–14·0	0·106
1st tertile	4·4	1·8	4·3	1·9	0·216	4·5	1·7	4·3	1·9	0·209
2nd tertile	9·8	1·5	9·7	1·6	0·333	10·1	1·5	9·7	1·6	0·017
3rd tertile	18·6	6·2	19·0	7·2	0·393	18·7	6·2	18·9	7·1	0·758
Total added sugars, % total energy	5·8	3·1–9·2	5·5	2·8–9·3	0·214	6·1	3·3–9·3	5·5	2·8–9·3	0·094
1st tertile	2·0	1·1–2·9	2·0	1·1–2·8	0·608	2·33	1·5–3·0	1·9	1·1–2·9	0·032
2nd tertile	5·6	1·2	5·6 (1·2		0·819	5·5	1·1	5·6	1·2	0·349
3rd tertile	23·3	6·9	12·9	5·2	0·545	13·2	8·0	13·0	5·3	0·225
Fructose, % total energy	2·0	0·8–3·9	1·8	0·7–3·5	0·007	2·28	0·93–3·98	1·8	0·7–3·6	0·005
1st tertile	0·5	0·2–0·8	0·5	0·2–0·7	0·522	0·44	0·25–0·81	0·5	0·2–0·7	0·678
2nd tertile	1·9	0·5	1·9	0·6	0·950	1·96	0·53	1·9	0·5	0·622
3rd tertile	4·5	3·6–6·3	4·7	3·6–6·4	0·771	4·4	3·6–6·5	4·7	3·7–6·4	0·426
Total energy, kcal per day	1855	905	1936	1028	0·152	1837	896	1927	1015	0·415

FGID, functional gastrointestinal disorders; IBS, irritable bowel syndrome.

*P* values are based on two-sample Wilcoxon rank-sum (Mann–Whitney) test, two-sample *t* test or *χ* ^2^ test, based on survey design and test the equality of means within continuous variables by manifestation or not of at least one FGID symptom or IBS or whether there is statistically significant relationship between categorical variables and FGID or IBS category based on Rome III diagnostic questionnaire or (*n* 326) and HNNHS (*n* 3630).

Raw *P* values are provided. Type I error following Bonferroni correction, at 5 % level, is n/0·05, which is approximately < 0·0007; a *P* value < 0·001 is not precise to conclude.

* Variables presented as percentage to the total energy consumption following the median (25th–75th percentile) or mean (sd) scheme according to each variable’s distribution. Additionally, variables referring to total sugars’ consumption are also divided in tertiles in favour of better display.

†Tests equality of variances within shown daily consumption variables, by manifestation or not of at least one FGID symptom (*P*-value1).

‡ Tests equality of variances within shown daily consumption variables, by manifestation or not of IBS symptoms (*P*-value2).

**Fig. 2.**
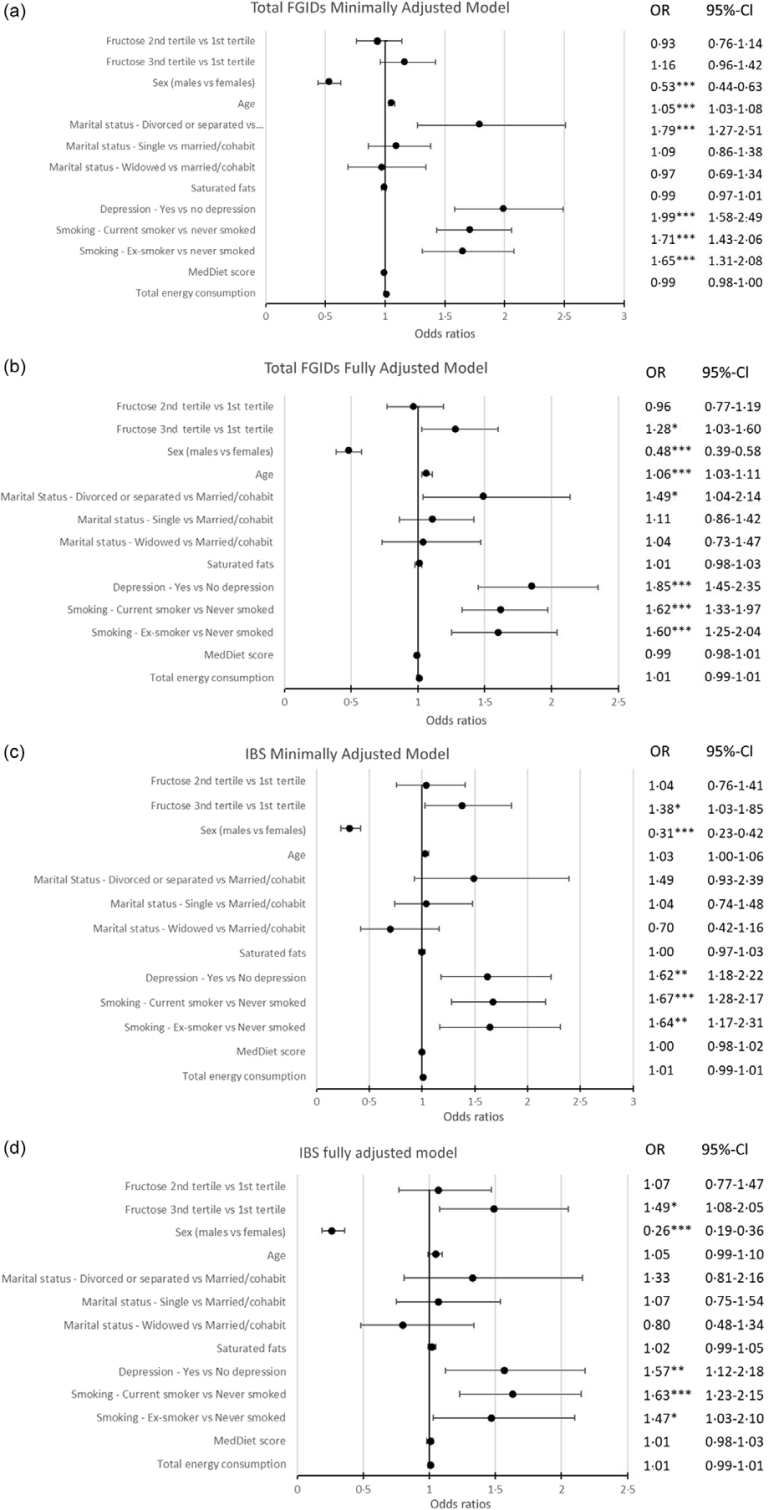
Forest plot of multiple logistic regression model for FGID and IBS likelihood. (a) and (c): Model adjusted for age and sex (minimally adjusted model, (a) for FGID and (c) for IBS). (b) and (d): Model adjusted for fructose consumption, sex, age (per 5-year increase), marital status, saturated fats intake, depression, smoking status, MedDiet score categories and energy intake (fully adjusted model, b for FGID and d for IBS). Significance set at alpha = 5 %. Level of significance *P < 0·05, **P < 0·01, ***P < 0·001. Fructose tertiles: % energetic contribution of fructose consumption to the total energy consumption divided in tertiles (Q2 and Q3 compared with Q1). Raw *P* values are provided. Type I error following Bonferroni correction, at 5 % level, is n/0·05, which is approximately < 0·0007; a *P* value < 0·001 is not precise to conclude. FGID, functional gastrointestinal disorder; IBS, irritable bowel syndrome.

Finally, in Fig. 2, the results of the minimally and fully adjusted logistic regression models, for those with FGID compared with those without, and those with IBS specifically, in relation to tertile of fructose intake, are depicted. The covariates used in the minimally adjusted model were age and sex and in the fully adjusted models were age (per 5-year increase), sex, marital status, saturated fats consumption, depression, smoking status, MedDiet score categories and energy intake, along with fructose consumption tertiles. These covariates were all found to be weakly (0·2 ≤ *r* ≤ 0·39) or very weakly correlated (0 ≤ *r* ≤ 0·19) to each other, and the mean variance inflation factor value was lower than 5·06 for all models used in the analyses, indicating the absence of multicollinearity between variables^(48–50)^. Overall, FGID likelihood was 1·28 times higher in individuals in the higher fructose consumption tertile (Q3) compared with the lowest (Q1), in the fully adjusted model (OR 1·28; 95 % CI: 1·03, 1·60); no association was found in the minimally adjusted. For IBS, the likelihood was higher in both models with the likelihood increasing from 1·38 times in the minimally adjusted to 1·49 times in the fully adjusted model among those with higher fructose intakes (OR 1·38:95 % CI: 1·03, 1·85 and OR:1·49; 95 % CI: 1·08, 2·05, respectively). Other factors that were included and were independently associated with the outcome included sex, depression, marital and smoking status. Being male, having no reported depressive symptoms, being married and never having smoked were associated with lower likelihood for any FGID manifestation (protective). The same was observed for IBS excluding age which had no significant effect. Furthermore, increased MedDiet score adherence decreased likelihood for IBS. The odds ratios for the likelihood of any FGID or IBS manifestation by minimally and fully adjusted model are presented in Fig. 2.

**Fig. 3.**
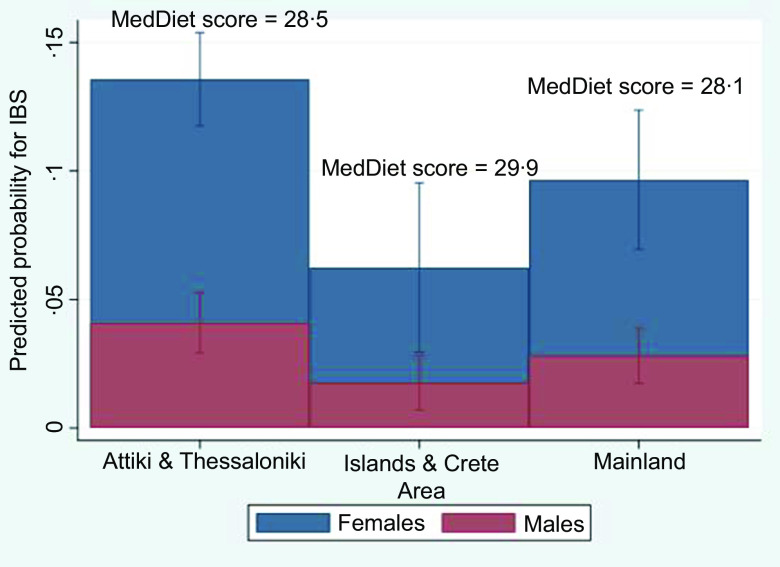
Predicted probability of IBS by area of residence in males and females, with information on mean MedDiet score by area. IBS, irritable bowel syndrome.

The prediction probability for IBS by area (Attiki % Thessaloniki, Mainland, and Islands & Crete), stratified by sex, (Fig. 3) revealed lowest levels among islanders (6 % in islanders compared with 9·5 % among mainlanders and 13 % in main metropolitan areas) for both males and females, with females having a higher probability in all areas compared with men. Post hoc analysis showed a significant higher MedDiet score and a significant lower intake of added sugar (% energy) among Islanders, compared with those of other areas (added sugar intake: 5·1 % (25th–75th percentile: 2·5–9·2 %) compared with 5·7 % (25th–75th percentile: 3·0–9·4 %), respectively). Fructose intake distribution (median–range) did not differ between individuals residing in the islands and the metropolitan areas (0·7–3·7 % in islanders and 0·8–3·7 % in individuals from the main metropolitan areas).

## Discussion

The main finding of the present study is that dietary fructose intake was associated with increased likelihood of any FGID and IBS specifically, irrespective of sex, depressive symptomatology and lifestyle habits, only among adults with higher intakes in relation to total energy consumption. In addition, it was also observed that one in five of the adult population experienced at least 1 FGID condition previously diagnosed by a physician, representing approximately 1·8 million Greek adults, 40 % of whom had IBS. FGID affected mostly females residing in main Metropolitan areas and separated or divorced adults and those of higher education.

The findings are of importance since worldwide FGID affects an average 30 % of the adult population residing in Western Countries, with a 20–50 % attributed to IBS^(2)^. Moreover, 40 % of the global population has been found to have suffered from an FGID at some point in their lives causing a great burden for their countries’ economies and health systems and having a great impact on each individual’s quality of life^(5,51)^. It is noteworthy to mention that healthcare costs for patients with IBS are estimated to $2 billion per year for China and £45·6–£200 million per year in the UK^(52)^. This is an area that needs to be addressed since in the present study, a high prevalence of FGID was observed, especially among individual was related with those with higher fructose intake.

It has been proposed that high amounts of fructose intake or high concentrations can disrupt metabolic processes and trigger organ malfunction and as a result can contribute to FGID symptoms^(14,53)^. It is noteworthy that only 50 % of healthy individuals can fully absorb 25 g of fructose as a 10 % solution causing large variations in how high fructose consumption is translated at an individual level^(54,55)^. Unabsorbed free fructose can react with incompletely digested proteins and may form advanced glycation end products (dAGEs) in the intestine^(56–58)^. It has been hypothesised that these dAGEs are associated with inflammatory diseases and gastrointestinal, respiratory and tissue distress,^(59,60)^ and it has been proposed that these products may trigger the mechanism behind these malfunctions. In addition, recent studies have included potential genetic factors to fructose malabsorption. For example, the carbohydrate response element-binding protein might play a possible role in fructose metabolism and tolerance, further adding to the variation of potential malabsorption^(55,61)^. The complexity of the involved mechanisms and the inherent uncertainty has led to the suggestion that fructose restriction may be a dietary solution for IBS symptomatology and relief from FGID symptoms^(16,62–64)^, although a paucity remains in the literature. Based on these reactions and potential fructose-malabsorption mechanism, the fructose concentration of processed food in the market and widely consumed should also be considered. Fructose content varies among sweetened beverages and processed foods in general, with a beverage of 100 % apple juice having an average fructose to glucose ratio of 2:1^(56)^, but commonly remains higher per portion than the amount found in fruits and vegetables^(35)^. Natural foods are characterised by small amounts of fructose, at an average level of about 5–8 % of their weight (e.g. 4·05 % fructose content in pineapples and 8·65 % in green grapes) accounting sometimes for free fructose to glucose excess^(65)^. For example, distinctive fruits providing unpaired fructose are apples (∼4·3 g EFF/medium-sized apple), pears (∼5·9 g EFF/medium-sized pear), mangoes (∼4·4 g EFF/medium-sized mango), watermelon (∼2·8 g EFF/1 diced 8-oz cup) and green grapes (∼1 g EFF/100 g)^(35)^. In any case, when consuming raw natural foods that contain fructose, this is delivered along with water, fiber, antioxidants and various other whole food constituents which combined result to a slow gastrointestinal absorption following consumption and a minor increase in circulating fructose^(35,66)^.

Expanding on the effects that fructose source may have, the potential preventive effect of the Mediterranean diet on FGID should also be considered. The present study showed that although the likelihood of any FGID was associated with higher fructose intake, the predicted probability decreased with higher adherence to the Mediterranean diet. In particular, Islanders had a lower probability of IBS and higher adherence to the Mediterranean diet compared with the individuals residing in the main Metropolitan areas and the mainland. The association of fructose with increased FGID symptomatology may therefore be related to fructose intake which is ingested mainly from processed food. Although this cannot be directly revealed from this analysis, it is recommended that future studies separately address natural *v*. processed fructose sources, based on this study’s observations. It is noteworthy in the past several years, the traditional Mediterranean diet in Greece is transitioned to a more Westernized one,^(21,46)^ and this may partly explain the association observed between FGID and higher fructose intake.

Another explanation of the association between fructose consumption and FGID could be the potential malabsorption observed with high dietary fructose intakes mostly from processed foods, as other studies have reported^(16,67)^. Specifically, higher free fructose intake could lead to lower abundance of beneficial microbes for carbohydrate metabolism^(68)^ in the gut microbiota and may trigger general gastrointestinal discomfort, including that of IBS, due to its slow absorption leading to increased osmotic load and fermentation, especially in the presence of visceral hypersensitivity^(12,69,70)^. The specific mechanisms, however, have not been addressed by this study.

Other factors from our study that were found to be correlated with FGID, include age, sex, smoking, PA, sleep disturbances, stress and other psychological factors^(2)^. The results of our study are in accordance with the results from another study, conducted in Mexico, which observed that women were at a 50 % higher risk of having an FGID^(71)^. The correlation of sex and FGID symptomatology was observed, along with other factors, in other studies too^(52,72)^. A very interesting study of 27 949 French adults from the general population reported that IBS patients were more likely to be current smokers, younger, single, with low income and following a healthier diet^(73)^. Regarding stress, depression and sleeping quality, a recent study by Hwang S-K and co-workers indicated significant associations between depression, anxiety, stress and bad sleeping quality with the severity and occurrence of FGID symptoms^(74)^.

### Limitations and strengths

Results must be interpreted with caution since ROME III questionnaire, although used as a screening tool in epidemiological studies, it was not designed to detect structural disorders and abnormalities, hence alone is an insufficient tool for diagnosis. Another limitation includes the potential for Type I error due to the multiple tests that were performed, despite the associations found in the adjusted analysis. Results must therefore be carefully interpreted s; a *P* value of < 0·001 is not precise to conclude. Also, this study is of a retrospective design with both exposure and outcome being assessed simultaneously and, therefore, does not allow to extract true causal effects to the outcome (total FGID and IBS symptomatology). Longitudinal data could upgrade the power of the current study’s results as well as the examination of the type of fructose (unpaired, paired free and paired in sucrose), in relation to FGID/IBS. Also, even though there was a significant difference in FGID and IBS prevalence between females and males, data analysis was not stratified by sex, due to the limited number of ROME III response rate with the long structure of the questionnaire being the main reason for the low response rate. The limited number of individuals in subgroup analysis led to a wide CI, and although a difference was detected, the true value may vary. This, however, does not compromise the strength of the study, which combined ROMEIII results with reported information. This study has many strengths, as it employed a validated questionnaire to assess presence of FGID symptoms. All individuals identified were aware of their status, and some who were not classified had reported having been previously diagnosed. This underlines the importance that multiple assessment methods to increase the sensitivity of identifying individuals with FGID symptoms. Another strength was that a national representative study was used to examine not only fructose but also its relation to the Mediterranean diet, a well-established dietary pattern. This is essential to differentiate whether overall high fructose intake may enhance FGID symptoms, and, specifically IBS, or fructose from other processed sources, in the context of a more Western type of diet.

### Conclusion

This study adds to the knowledge regarding the association between FGID symptomatology and fructose consumption, showing that the main determinants are largely modifiable, including high fructose intake in areas with lower Mediterranean diet adherence. Population-specific programmes examining specific contributing food sources to fructose consumption, to other dietary patterns, may help decrease the prevalence of FGID, and IBS specifically.
